# Spatial targeted vector control in the highlands of Burundi and its impact on malaria transmission

**DOI:** 10.1186/1475-2875-6-158

**Published:** 2007-12-03

**Authors:** Natacha Protopopoff, Wim Van Bortel, Tanguy Marcotty, Michel Van Herp, Peter Maes, Dismas Baza, Umberto D'Alessandro, Marc Coosemans

**Affiliations:** 1 Department of Parasitology, Prince Leopold Institute of Tropical Medicine, Nationalestraat 155, B-2000 Antwerp, Belgium; 2 Department of Animal Health, Prince Leopold Institute of Tropical Medicine, Nationalestraat 155, B-2000 Antwerp, Belgium; 3 Medical Department, Médecins Sans Frontières Belgium, 94 rue Dupré, Brussels, Belgium; 4 Programme de Lutte contre les Maladies Transmissibles et Carentielles, Ministry of Health, Bujumbura, Burundi; 5 Department of Biomedical Sciences, Faculty of Pharmaceutical, Veterinary and Biomedical Sciences, University of Antwerp, Universiteitplein 1, B-2610, Belgium

## Abstract

**Background:**

Prevention of malaria epidemics is a priority for African countries. The 2000 malaria epidemic in Burundi prompted the government to implement measures for preventing future outbreaks. Case management with artemisinin-based combination therapy and malaria surveillance were nationally improved. A vector control programme was initiated in one of the most affected highland provinces. The focal distribution of malaria vectors in the highlands was the starting point for designing a targeted vector control strategy. The objective of this study was to present the results of this strategy on malaria transmission in an African highland region.

**Methods:**

In Karuzi, in 2002–2005, vector control activities combining indoor residual spraying and long-lasting insecticidal nets were implemented. The interventions were done before the expected malaria transmission period and targeted the valleys between hills, with the expectation that this would also protect the populations living at higher altitudes. The impact on the *Anopheles *population and on malaria transmission was determined by nine cross-sectional surveys carried out at regular intervals throughout the study period.

**Results:**

*Anopheles gambiae *s.l. and *Anopheles funestus *represented 95% of the collected anopheline species. In the valleys, where the vector control activities were implemented, *Anopheles *density was reduced by 82% (95% CI: 69–90). Similarly, transmission was decreased by 90% (95% CI: 63%–97%, p = 0.001). In the sprayed valleys, *Anopheles *density was further reduced by 79.5% (95% CI: 51.7–91.3, p < 0.001) in the houses with nets as compared to houses without them. No significant impact on vector density and malaria transmission was observed in the hill tops. However, the intervention focused on the high risk areas near the valley floor, where 93% of the vectors are found and 90% of the transmission occurs.

**Conclusion:**

Spatial targeted vector control effectively reduced *Anopheles *density and transmission in this highland district. Bed nets have an additional effect on *Anopheles *density though this did not translate in an additional impact on transmission. Though no impact was observed in the hilltops, the programme successfully covered the areas most at risk. Such a targeted strategy could prevent the emergence and spread of an epidemic from these high risk foci.

## Background

Malaria epidemics occur frequently in the African highlands [1-3]. Their control is a priority and a specific plan of action was adopted by the African leaders during the 2000 Abuja summit [4]. An early warning system to increase malaria epidemic preparedness and prevention has been promoted, based on climate data, population vulnerability indicators, environmental factors and disease surveillance [5]. Models proposed seems reliable in desert fringes [6,7], where rainfall is the main driving factor of epidemics [8]. However, the available forecasting models may not be accurate enough for the African highlands where most populations at risk of epidemics reside [8,9]. Consequently, in the highlands, routine implementation of preventive measures and prompt response to an unexpected increase of malaria cases are the main components for the control of epidemics.

In the last decade, Burundi has faced an increase in malaria cases with a major malaria outbreak in 2001 [10]. To contain the epidemic, Indoor Residual Spraying (IRS) and Long-Lasting Insecticidal Nets (LLIN) have been implemented in the highland province of Karuzi [11]. Due to its late implementation, this strategy was unable to have any impact on the epidemic. However, this experience showed that these interventions were feasible, even in the context of a complex emergency situation.

Following the 2001 epidemic, the national health authorities decided to improve data collection, adopted an interim treatment protocol based on artemether-lumefantrin only during malaria epidemics and started studies to change the national treatment policy for an artemisinin-based combination therapy (ACT). Furthermore, in Karuzi, one of the most affected areas, focal vector control activities were implemented. The objective of these measures was to prevent future malaria outbreaks.

In epidemic areas, the distribution of anopheline mosquitoes and malaria transmission are usually focal [12] and often negatively associated with distance from rivers or valley bottoms [13,14]. Therefore, rather than implementing vector control activities over large areas, it was felt that they could be targeted to places where most malaria transmission occurs, possibly reducing the implementation costs, and enhancing their sustainability without losing effectiveness [15]. Besides reducing transmission in the targeted valleys, it was thought that the hills above the IRS areas would also benefit as they would be shielded from the transmission occurring from below [16]. Similar approach, i.e. focal intervention based on vector behaviour, was successfully used to control malaria with environmental measures in the early 20^th ^century in Indonesia [17] but later abandoned in the DDT era. Since then, only one study addressed this issue for African highland [16]. This paper reports the results of such targeted intervention on vector density and malaria transmission in the Burundi highlands.

## Methods

### Study area

Karuzi is a central highland province in Burundi. A detailed description was presented elsewhere [11]. In 2002, malaria was the main cause of morbidity, representing 57% of all attendance to health facilities (Médecins Sans Frontières-Belgium malaria dataset, 2002). Malaria cases peak in June–July and in November–December. The recent epidemics in the Burundian highlands were mostly recorded towards the end of the year (EPISTAT: Epidemiology and Statistic Cell of the Ministry of Health, Burundi).

### Interventions and study

Between 2002 and 2005, an annual IRS round (June–July) was carried out targeting the areas at the foot of the hills before the second transmission period. The rationale of such choice was based on the observation that malaria vectors seldom spread further than one kilometre radius from the breeding sites [15,17]. In the highlands, vectors are usually clustered at the valleys' bottom from where they do not spread beyond 500 meters [18]. In this study only the valleys, i.e. the zone from the river/marsh at the bottom of the valley up to 700 meters uphill, were treated, while the upper part of the hills were left untreated. In the intervention areas, IRS was carried out in all human dwellings (interior walls and ceilings) and cattle sheds with the residual insecticides deltamethrin 5 WP (in 2002–2004) or alphacypermethrin 5 WP (in 2005) at the dose of 25 mg a.i./m^2^. In 2002, the LLIN (PermaNet^® ^1.0) distribution preceded the first IRS round and consisted of two nets for each sprayed house.

**Figure 1:**
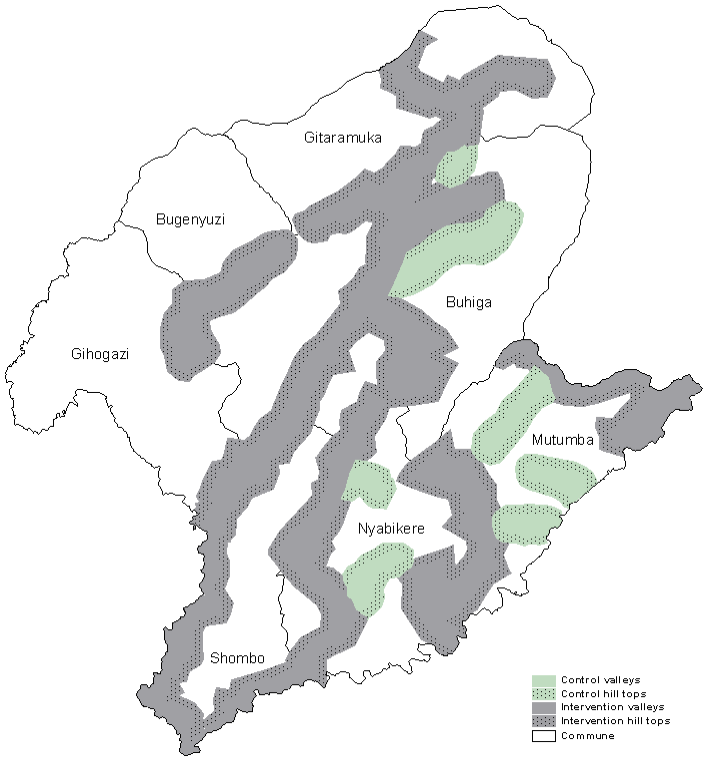
**Map of Karuzi Province (Burundi) showing the intervention and control areas**. In the intervention areas only the valleys were sprayed depicted in grey. The hill tops were not sprayed (dotted grey). The control areas are represented in green for the valleys and dotted green for the hill tops.

**Figure 2:**
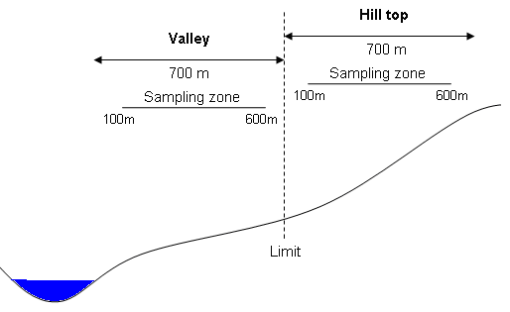
**Representation of the valley and hill top areas, showing the sampling zones**. From the valley floor, clusters in the valley where chosen at random between 100 and 600 metres. Clusters in the hill top were selected from 100 to 600 metres from the limit separating valley and hill top.

The larger cultivated valleys, with the highest population density, were chosen for the intervention, while other areas were identified as control areas (Figure 1). Because intervention areas were actually selected for their higher malaria risk and this for obvious ethical reasons, they are probably not entirely comparable to control areas. Antimalarial treatment was available for both intervention and control areas. The total length of both sides of the valley floors, control (74 kilometres) and intervention (331 kilometres) alike were equally divided into 100 points on a digital map of the province. For each survey, 25 points were randomly selected for both intervention and control area and their latitude and longitude sent to a hand-held global positioning system (GPS 76, Garmin^®^). From the geographical location of each selected point, two clusters consisting of either four or eight houses (according to the survey) where chosen; The valley clusters comprised houses located around a randomly chosen point on a vertical line running between 100 and 600 meters from the bottom; The hilltop clusters comprised houses located around a randomly chosen point on a vertical line running between 100 and 600 meters from the "limit" (700 metres from the valley bottom) separating valley and hill top (Figure 2). Hence, four zones were identified: (1) the intervention valleys with treated houses, (2) the corresponding intervention hill tops without treatment, (3) the untreated control valleys and (4) the untreated control hill tops. In total 4 × 25 clusters were re-sampled for each survey.

A baseline entomological survey (Survey 1) was done before the intervention and was followed for four years by two annual surveys: one three months (November–December: survey 2, 4, 6, 8) and the other nine months (April–May: survey 3, 5, 7, 9) after the yearly IRS. The types of houses (size, open eaves, walls and roof), presence of animals, location of the kitchen, altitude and distance from the selected cluster to the swamp were recorded, based on direct observation.

Daytime indoor resting mosquitoes were collected by spray-sheet catches using aerosol with pyrethrum and piperonyl butoxide [19]. These spray-sheet collections were done in randomly selected un-sprayed houses of the control areas and intervention hill tops. In the intervention valleys the collection was made regardless of the spraying status of the houses during the preceding IRS round. The Indoor Resting Density (IRD) was determined as the average number of *Anopheles *collected for each house.

Anophelines were morphologically identified and classified as *Anopheles gambiae *sensu lato (s.l.) and *Anopheles funestus *using a simplified key adapted from Gillies [20]. The feeding status (unfed, blood-fed, half-gravid and fully-gravid) was also scored. A sample of *An. gambiae *s.l. and *An. funestus *mosquitoes was analysed by species specific Polymerase Chain Reaction (PCR) [21,22]. The head and thorax of all collected females were individually tested with an enzyme-linked immunosorbent assay (ELISA) adapted from Wirtz [23] for the presence of *Plasmodium falciparum *circumsporozoite antigen. The sporozoite rate (SR) was computed as the proportion of ELISA positive mosquitoes. The number of infective bites per house per month was estimated as the number of fed *Anopheles *resting indoor and positive for *P. falciparum *by the ELISA test [24,25].

### Statistical analysis

Data were analysed using STATA software (Stata-Corporation, USA, version 9.2). In all statistics analyses, house clusters were taken into account to calculate robust standard errors and 95% confidence interval (95%CI). Logistic regressions were used to analyse the SR whereas negative binomial regressions were used for the counts (IRD and number of infective bites).

Baseline data on housing characteristics in the four zones were summarized by means of proportions or means. All entomological indicators, *Anopheles *species, *An. funestus *and *An. gambiae *IRD, SR and number of infective bites were analysed separately for the valleys and the hilltops data. *Anopheles *IRD was tested using the survey identification, intervention vs. control and the interaction between both as discrete explanatory variables. The density of *An. funestus *and *An. gambiae *in the hilltops were also computed using the seasons (before, three months or nine months after IRS), the intervention vs. control and their interaction terms as explanatory variables. SR and the number of infective bites were analysed for the dataset of surveys 2–9 pooled together with intervention vs. control as explanatory variables.

All entomological indicators were analysed in control areas for surveys 2–9 using hilltop vs. valley as only explanatory variable. Finally, the effect of the LLINs on the *Anopheles *IRD and on the number of infective bites was evaluated on the dataset restricted to the intervention valleys. Density Ratios (DR) were calculated as the exponential of the negative binomial regression coefficient for the IRD and for the number of infective bites.

### Ethical issues

Verbal consent was asked to the head of each household for the spray catches. In case of refusal (usually less than 10 by surveys) the next household was asked for permission. The vector control programme and the study were approved by the Ministry of Health of Burundi. Ethical approval for this study was granted by the Ethical Committee of the Institute of Tropical Medicine in Antwerp.

## Results

### Vector control activities

A total of 24,000 LLINs were distributed (Table 1). In 2002, just after the main distribution, most nets were in use (18792/23850, 78.8%), the rest being either not used (2632/23850, 11.0%) or missing. However, during the period 2002–2005, LLIN use decreased to 31.2%. IRS coverage exceeded 90%, except for the year 2002 (Table 1). During the first IRS round, 1,600 houses in the southern part of the province could not be sprayed because of security problems. These houses were treated and received LLINs the following years.

**Table 1:** Coverage of the vector control activities, Indoor Residual Spraying (IRS) and Long Lasting Insecticidal Net (LLIN) by year

	2002	2003	2004	2005
Total provincial population*	302 062	311 134	320 458	329 431
Nbr of targeted houses (% treated)	14 783 (86%)	15 106 (95%)	17 954 (93%)	18 072 (94%)
Nbr of houses treated/man/day	6.0	5.7	5.7	5.4
Insecticide used	Deltamethrin	Deltamethrin	Deltamethrin	Alpha cypermethrin
Nbr of LLIN distributed	20 750	3 200	0	0
Nbr of net used (%**)	18 792 (78.8%)	17 631 (65.2%)	14 442 (53.4%)	8 431 (31.2%)

* Official data (EPISTAT Burundi)

** % = net used/(LLINs distributed + 3100 net present in the houses before the intervention)

### Baseline characteristics of households and malaria transmission

Before the intervention, i.e. survey 1, the house characteristics between intervention and control areas were similar (Table 2), except for the clusters in the hill tops of the intervention areas which were more distant from the valley bottom (1,216 metres) than those in the control areas (945 metres). The separation between valleys and hill tops was chosen at 700 meters from the valley bottom. However, in the intervention areas during the houses census, the limit was moved further away in some areas and explained the difference between clusters in the hill tops. In the subsequent surveys, no major differences between control and intervention areas in terms of house type (size, open eaves, walls and roof), domestic animals, location of the kitchen, mean altitude of the clusters and distance from valley clusters to valley bottom could be found.

**Table 2:** Environmental and household characteristics in the intervention (I) and control (C) areas for pre-intervention survey (survey 1).

	Valleys		Hill tops	
		
	C	I	C	I
No. of houses sampled	100	100	99	150
Houses with animals inside	68.0 (4.5)	62.0 (6.1)	69.4 (5.1)	64.7 (6.0)
Open eaves	57.0 (5.1)	49.0 (6.0)	48.5 (6.0)	47.3 (5.7)
Separate kitchen	22.0 (4.4)	19.0 (4.6)	20.2 (4.8)	22.0 (6.0)
Size of houses				
*< 25 m*^2^	11.0 (4.6)	28.0 (5.6)	19.2 (5.5)	19.3 (3.7)
*25–50 m*^2^	19.0 (4.6)	22.0 (5.3)	21.2 (4.3)	34.0 (4.2)
*> 50 m*^2^	70.0 (6.5)	50.0 (7.6)	59.6 (6.5)	46.7 (5.5)
Type of walls				
*Thatch*	2.0 (1.4)	10.0 (3.8)	8.1 (3.8)	8.0 (2.7)
*Mud*	69.0 (7.0)	54.0 (7.5)	67.7 (6.7)	61.3 (6.8)
*Bricks*	24.0 (6.0)	33.0 (6.6)	19.2 (4.2)	25.3 (5.4)
*Other*	5.0 (2.0)	3.0 (1.7)	5.1(2.1)	5.3 (2.5)
Type of roofs				
*Thatch*	51.0 (7.1)	53.0 (8.5)	52.5 (6.2)	55.3 (7.1)
*Tile*	14.0 (4.6)	16.0 (5.4)	19.2 (5.2)	12.0 (3.7)
*Corrugate*	19.0 (5.1)	22.0 (6.2)	23.2 (4.4)	27.3 (6.5)
*Other*	16.0 (4.8)	9.0 (3.2)	5.1 (2.9)	5.3 (1.9)

Altitude clusters (m)	1554 (11.9)	1548 (12.0)	1599 (18.4)	1607 (14.3)
Distance clusters/valley floors (m)	387(27.5)	404 (29.0)	945 (39.8)	1216 (71.5)

Proportions (standard error) are reported except for altitude and distance where arithmetic means (standard error) are given.

Before the intervention, malaria transmission in the valleys was significantly higher in the intervention than in the control valleys, mainly because of differences in *Anopheles *density and not sporozoite rates (Table 3). However, the hilltops of both intervention and control areas were comparable in term of transmission and *Anopheles *density.

**Table 3:** Baseline Indoor Resting Density (IRD), Sporozoite Rate (SR) and infective bites as observed during the pre-intervention survey in Control (C) and Intervention (I) areas.

	Valleys				Hill tops			
		
	C	I	Ratio* (95% CI)	P value	C	I	Ratio* (95% CI)	P value
IRD total *Anopheles*/house	1.3	7.6	5.9 (1.7–21.0)	0.007	1.3	0.6	0.4 (0.1–1.6)	0.200
SR total *Anopheles *(No. tested)	3.8% (104)	4.2% (737)	1.1 (0.4–3.1)	0.858	0.9% (114)	6.3% (64)	7.5 (2.2–26.2)	0.002
IRD fed *Anopheles*/house	0.5	4.3	8.7 (2.3–32.9)	0.002	0.4	0.2	0.6 (0.2–1.8)	0.333
SR fed *Anopheles *(No. tested)	2.0% (49)	4.1% (419)	2.0 (0.4–11.0)	0.397	2.8% (36)	9.4% (32)	3.6 (0.6–22.3)	0.156
Infective bites/house/month	0.3	5.1	17.0 (1.7–171)	0.015	0.3	0.6	2.0 (0.2–23.3)	0.580

*Density ratios for indoor resting density and infective bites. Odd ratios for sporozoite rates

### Entomological results

#### Species composition

A total of 18,764 mosquitoes were collected indoors, 77.1% (14,474) anophelines. *Anopheles gambiae *s.l. and *An. funestus *were the most abundant species (up to 95% of catches), with females *An. gambiae *s.l. (9473, 79.3%) more prevalent than *An. funestus *(2471, 20.7%), except for survey 1 where 57.6% of *Anopheles *were *An. funestus*. *Anopheles gambiae *s.s. (98.2%) was the dominant species of the complex. A few *Anopheles arabiensis *(60, 1.8%) were collected, most of them in April–May 2004 and 2005. Within the *Anopheles *species morphologically identified as *An. funestus *(n = 1898), 79.3% were *An. funestus *s.s. by species specific PCR [22]. For the remaining samples, the PCR and the sequencing analysis of the ITS2 region revealed no link with recorded species. After careful morphological identification, they could be identified as *Anopheles demeilloni *(Ralph Harbach personal communication), for which no sequence exists. This species is morphologically close to the *An. funestus *group and could not be separated using simplified identification keys. It will be further defined as "*Anopheles funestus-*like".

#### Indoor resting density

After the intervention, the overall reduction of *Anopheles *density in the valleys was 82.5% (95% CI: 69.4–90.0, p < 0.001) in the intervention compared to control areas. This significant difference was observed for every survey done three or nine months after IRS (Table 4). After the intervention, *Anopheles *density in the hilltops was only significantly reduced in the intervention for surveys 5 and 6 (Table 4). A lower density of *An. funestus *was only observed for the surveys done three months after the spraying (DR: 0.45, 95% CI: 0.25–0.81, p = 0.008), while for the others the difference was not statistically significant (DR: 0.66, p = 0.199). A similar DR was observed in *An. gambiae *s.l. (DR: 0.46, 95% CI: 0.15–1.41, p = 0.174) three months after IRS. However, the high intraclass correlation of the latter data caused an important design effect (Deff = 4.3 compared to 1.7 obtained in the analysis of *An. funestus *data) and reduced the power of the statistical analysis.

**Table 4:** Mean indoor resting density per house of all *Anopheles *in valleys and hill tops of intervention (I) and control (C) areas. Differences by survey were tested with the negative binomial regression.

	Valleys				Hill tops			
		
Survey	C	I*	Density ratio (95% CI)	P value	C	I*	Density ratio (95% CI)	P value
2	3.26	0.13	0.04 (0.01–0.13)	<0.001	1.03	0.39	0.37 (0.13–1.05)	0.061
3	1.81	0.27	0.15 (0.06–0.40)	<0.001	0.34	0.74	2.20 (0.87–5.58)	0.096
4	1.87	0.18	0.09 (0.03–0.26)	<0.001	0.65	0.32	0.49 (0.23–1.02)	0.055
5	7.12	0.52	0.07 (0.03–0.16)	<0.001	3.36	0.90	0.27 (0.12–0.61)	0.002
6	2.51	0.27	0.11 (0.02–0.70)	0.020	0.76	0.18	0.24 (0.09–0.64)	0.004
7	8.70	3.44	0.40 (0.16–0.95)	0.039	2.18	3.39	1.56 (0.56–4.30)	0.392
8	11.80	1.19	0.10 (0.03–0.32)	<0.001	3.13	1.76	0.56 (0.15–2.07)	0.386
9	3.58	1.15	0.32 (0.15–0.70)	0.004	1.53	1.73	1.13 (0.34–3.84)	0.839

*Areas initially selected as intervention and not sprayed during the first year were not included in the analysis of survey 2 and 3.

Odd surveys: April–May, 9 months after the annual IRS round

Even surveys: November–December, 3 months after the annual IRS round

In the intervention valleys, an additional protective effect due to LLINs was observed, with a decrease of *Anopheles *density of 79.5% (95% CI: 51.7–91.3), p < 0.001) in the November–December surveys pooled together. The LLINs were given to protect the population during the May-June transmission season when the residual activity of the insecticide use for IRS, had ceased. However, nine months after IRS, a 56.2% reduction in *Anopheles *density associated with LLIN use narrowly missed statistical significance (95% CI: 0–71.0, p = 0.053).

#### Malaria sporozoite infection rates (SR) in Anopheles

SR was estimated for all specimens, regardless of their physiological status. Before the intervention, the *P. falciparum *SR was 6.2% (27/433) for *An. gambiae *s.l. and 2.2% (13/586) for *An. funestus*. The post-intervention SR (all surveys pooled together) was 1.0% (10/1018) in the intervention valleys and 2.4% (149/6235) in the control valleys (OR: 0.41, 95% CI: 0.22–0.74, p = 0.004). However, the difference was significant only for *An. gambiae *s.l. (OR: 0.39, 95% CI: 0.21–0.75, p = 0.004) and not for the morphological identified *An. funestus *(OR: 0.49, 95% CI: 0.06–3.80, p = 0.493), probably because of the limited number of specimens collected. On the hilltops, no significant difference in SR between control and intervention areas was observed (OR: 0.81, 95% CI: 0.36–1.81, p = 0.605). For the "*An. funestus*-like" species, ELISA tests (459) were negatives for all surveys.

#### Infective bites by house per month

**Figure 3:**
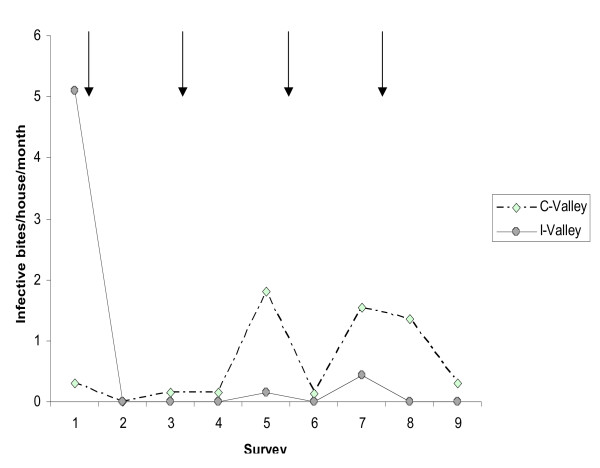
**Mean number of infective bites per house by survey in intervention and control valleys**. Arrows represent the spraying round. To estimate the transmission only freshly fed females positive with the ELISA test were considered.

In the valleys, vector control reduced the infective bites/house/month by 89.6% (95% CI: 62.5–97.1, p = 0.001). The number of infective bites was undetectable in the intervention valleys (Figure 3), except for surveys 5 and 7, where transmission increased but was still lower than in control valleys with DR of 0.08 (95% CI: 0.01–0.74, p = 0.026) and 0.28 (95% CI: 0.06–1.36, p = 0.114) respectively. In the intervention areas (valleys and hill tops), the transmission was reduced by 84.4% (95% CI: 60.1–93.9, p < 0.001) compared to control areas. No effect of LLIN-use was observed on transmission for the surveys performed three or nine months after the yearly IRS round. Sporozoite rates in fed anopheles used to calculate the number of infective bites were null in both treated houses with nets and without nets in the surveys done three months after the activities.

#### Comparison between valleys and hill tops

In survey 1, there was no difference for *Anopheles *density and infective bites/house/month between the valleys and the hilltops in control areas; in the intervention areas 93.1% of the *Anopheles *(95% CI: 78.9–98.1) and 89.6% of the malaria transmission (95% CI: 56.8–98.2) were found in the houses within 700 meters of the valley bottom. These differences were not seen in the intervention areas after the implementation of vector control activities. In control areas, from survey 2 to 9, higher densities of *Anopheles*, sporozoite rates and transmission were found in the valleys compared to the hill tops (Table 5).

**Table 5:** Comparison of entomological outcomes (indoor resting densities, sporozoite rates and infective bites) between valleys and hill tops of control areas (survey 2 to 9 pooled together).

	Valleys	Hill tops	Ratio* (95% CI)	P value
IRD total *Anopheles*/house	5.2	1.7	3.1 (1.9–5.2)	<0.001
SR total *Anopheles *(No. tested)	2.4% (6235)	1.2% (1898)	2.0 (1.1–3.7)	0.029
IRD fed *Anopheles*/house	1.3	0.5	2.5 (1.3–4.6)	0.005
SR fed *Anopheles *(No. tested)	2.0% (1812)	0.8% (714)	2.4 (0.9–6.1)	0.067
Infective bites/house/month	0.7	0.1	6.0 (2.2–16.8)	<0.001

*Density ratios for Indoor Resting Density (IRD) and infective bites. Odd ratios for Sporozoite Rates (SR).

## Discussion

Vector control based on IRS and insecticide-treated nets are effective tools in preventing malaria in the highlands [26,27]. In order to improve the cost effectiveness of such methods they could be targeted to the malaria high risk areas. Malaria transmission in African Highlands is often focal [28] and breeding sites are usually more common in the valley floors as seen in highlands of Kenya [29], Tanzania [16] and Rwanda-Burundi [13].

In Burundi, from 2002 to 2005, vector control measures combining IRS and LLINs were implemented in the highland province of Karuzi. These activities were spatially and timely targeted to enhance the feasibility and lower the cost. One round of IRS per year was organized in June–July before the seasonal increase in transmission. Moreover, only the valley floors, where most *Anopheles *breeding sites are, were treated. The activities were successfully implemented and the high coverage for IRS in the targeted areas has been sustained due to the strong support of the local authorities. The number of LLINs retained after distribution among targeted households was rather high for a low income population, no past history of net use and with low mosquito nuisance. However, the life span and fabric integrity of PermaNet^® ^in these poor housing settings was drastically reduced and coverage decreased quickly after the first year, mostly because net were holed by wood sticks and rats and thrown away; some of them were stolen. It appears then that, besides their insecticidal properties, LLINs should also be resistant enough to support hard field condition as those occurring in Burundi [30].

This study confirmed that most malaria transmission occurred close to the valley bottoms where rivers, marshes and agricultural activities are. In the intervention areas and before any vector control activity, 93% of *Anopheles *was found near the valley floors, a result consistent with the 98% found by Githeko in the Kenyan highlands [29]. In contrast to what was seen in the Tanzanian mountains [16], the expected protective effect of the treated valleys on the hill tops could not be demonstrated, except for *An. funestus *just after the yearly IRS rounds. This may suggest that the *Anopheles *density in the hilltops, particularly that of *An. gambiae *s.l., may depend also on local and higher breeding places.

Non-treated sentinel houses are commonly used to evaluate a mass effect on the vector population. However, in this study houses in the intervention valleys were selected at random, regardless of their spraying status during the previous IRS round. This method may provide a more representative picture of the real exposure of the human population. Human landing collections would have been more appropriate to estimate the transmission but this was not feasible because of insecurity. Collection of indoor resting mosquitoes is an alternative when considering the high endophily of malaria vectors in the highlands. The estimation of transmission intensity is then based only on freshly fed females positive for the circumsporozoite antigen by unit of time (month) [24,25].

This spatial targeted intervention drastically reduced the vector populations of *An. gambiae *s.l. and *An. funestus *in the treated valleys compared to the control valleys. By only spraying the valleys, malaria transmission was reduced by 89.6% in the targeted valleys and by 84.4% in the whole intervention areas. Moreover, because the control valleys had a significant lower *Anopheles *density and malaria transmission than intervention area in the baseline survey, the impact of the control measures may be underestimated.

From the fourth year of intervention (year 2005), a lower effect of the control activities on *An. gambiae *s.l. density was observed in the treated areas. This could be attributed to different factors: an overall *Anopheles *increased observed also in the control valleys, a lower quality of the spraying, the use of a different pyrethroid insecticide (alpha-cypermethrin) during the last year and the decreased used and/or efficacy of the LLINs. Finally, the repetitive used of IRS could have also selected pyrethroid resistant as recently shown for pre-impregnated plastic sheeting [31]. In Karuzi, an increase in *kdr *allele frequency, involved in pyrethroids resistance in *An. gambiae *s.s. was observed after each spray round and the importance of insecticide resistance would be further investigated. Pyrethroids resistances could hamper malaria control as observed in South Africa [32] and Equatorial Guinea [33].

Usually, IRS or LLIN's are implemented alone. Recently operational research, to determine the efficacy of combining both interventions areas, has been advocated by the WHO Global Malaria Programme [34]. In the Burundian context, LLIN-use confers an added value to IRS in reducing the *Anopheles *density in the houses. However, the high coverage achieved with IRS had already decreased the sporozoite rate to undetectable level and no additional reduction on transmission could be observed where LLIN are used.

## Conclusion

Vector control activities of IRS targeting valleys in highland were very effective in reducing *Anopheles *density and malaria transmission. These valleys are responsible for 90% of the transmission occurring in the area. In sprayed areas, LLINs reduced further the *Anopheles *density but not the transmission. Unfortunately, treating the valleys did not confer protection for adjacent hilltops, although the density of mosquitoes was much reduced there. Given limited resources, it appears that such targeted approach in highlands could avoid the spread of epidemic from these foci preventing outbreaks in the whole province.

## Authors' contributions

NP was involved in the study design, conducted the implementation of the operational activities and the data collection, performed statistical analysis and interpretation and drafted the manuscript. WVB was involved in interpretation of the data, helped to draft and revised the paper for intellectual content. TM and MVH were involved in the data analysis and revised the manuscript. PM initiated this program and revised the manuscript. BD was involved in the operational activities, the data collection and revised the manuscript. MC was responsible for the study design and conception. UDA contributed to the study design and with MC critically reviewed the manuscript for intellectual content. All authors have read and approved the final version of the manuscript.
